# Using human centered design to identify opportunities for reducing inequities in perinatal care

**DOI:** 10.1186/s12913-021-06609-8

**Published:** 2021-07-20

**Authors:** Malini A. Nijagal, Devika Patel, Courtney Lyles, Jennifer Liao, Lara Chehab, Schyneida Williams, Amanda Sammann

**Affiliations:** 1 Department of Obstetrics, Gynecology and Reproductive Sciences, UCSF/ZSFG, 1001 Potrero Avenue, Building 5, 6D-9, San Francisco, CA 94110 USA; 2 Department of Surgery, University of California, San Francisco, USA; 3 Center for Vulnerable Populations, University of California, San Francisco at San Francisco General Hospital, San Francisco, USA; 4 Department of Emergency Medicine, Jefferson University, Philadelphia, USA

**Keywords:** Human-centered design, Perinatal care

## Abstract

**Background:**

Extreme disparities in access, experience, and outcomes highlight the need to transform how pregnancy care is designed and delivered in the United States, especially for low-income individuals and people of color.

**Methods:**

We used human-centered design (HCD) to understand the challenges facing Medicaid-insured pregnant people and design interventions to address these challenges. The HCD method has three phases: Inspiration, Ideation, and Implementation. This study focused on the first and second. In the Inspiration phase we conducted semi-structured interviews with a purposeful sample of stakeholders who had either received or participated in the care of Medicaid-insured pregnant people within our community, with a specific emphasis on representation from marginalized communities. Using a general inductive approach to thematic analysis, we identified themes, which were then framed into design opportunities. In the Ideation phase, we conducted structured brainstorming sessions to generate potential prototypes of solutions, which were tested and iterated upon through a series of community events and engagement with a diverse community advisory group.

**Results:**

We engaged a total of 171 stakeholders across both phases of the HCD methodology. In the Inspiration phase, interviews with 23 community members and an eight-person focus group revealed seven insights centered around two main themes: (1) racism and discrimination create major barriers to access, experience, and the ability to deliver high-value pregnancy care; (2) pregnancy care is overmedicalized and does not treat the pregnant person as an equal and informed partner. In the Ideation phase, 162 ideas were produced and translated into eight solution prototypes. Community scoring and feedback events with 140 stakeholders led to the progressive refinement and selection of three final prototypes: (1) implementing telemedicine (video visits) within the safety-net system, (2) integrating community-based peer support workers into healthcare teams, and (3) delivering co-located pregnancy-related care and services into high-need neighborhoods as a one-stop shop.

**Conclusions:**

Using HCD methodology and a collaborative community-health system approach, we identified gaps, opportunities, and solutions to address perinatal care inequities within our urban community. Given the urgent need for implementable and effective solutions, the design process was particularly well-suited because it focuses on understanding and centering the needs and values of stakeholders, is multi-disciplinary through all phases, and results in prototyping and iteration of real-world solutions.

**Supplementary Information:**

The online version contains supplementary material available at 10.1186/s12913-021-06609-8.

## Background

Poor pregnancy outcomes and disparities in the United States are a sign of low-value and ineffective care. Despite spending more for care during pregnancy and childbirth, the United States achieves significantly worse outcomes with rates of maternal mortality, severe maternal morbidity (SMM), preterm birth and infant mortality among the highest of any developed country [1, 2]. The structure and content of outpatient prenatal and postpartum care largely emerged from medical opinion and tradition, rather than evidence tying it to better outcomes [3]. In addition, there is abundant evidence that structural, institutional, and interpersonal racism is deeply embedded into U.S. medical care, especially within obstetrics and gynecology [4–6]. These issues highlight the need to transform how pregnancy care is designed and delivered, especially for low-income individuals and people of color who face the worst inequities in pregnancy care access, experience, and outcomes.

Barriers to ineffective pregnancy care for low-income people are well-documented. Inadequate transportation, limited clinic hours that require time off from work, job insecurity, and short grace periods before being considered a “no-show” make it challenging to consistently attend prenatal care appointments [7, 8]. Standard prenatal visits, often 10–15 min in duration, usually focus on a pre-determined list of screening tests and questions, rather than prioritizing pregnant persons’ concerns and questions [9]. Psychosocial, educational, and resource support are often delivered through separate providers and programs-- requiring more of the pregnant person’s time, additional screening and intake processes, and a more fragmented care experience [10]. People of color experience widespread racism and discrimination during pregnancy care encounters, eroding the ability to trust and value the care being provided [4, 11]. While models such as Centering Pregnancy® and home visiting programs have been developed to overcome some of these barriers, disparities in care access, experience, and outcomes persist, even in communities where these novel programs have been implemented [12, 13]. To address these disparities, it is important to focus on the care experience of those facing the worst outcomes, and to design solutions accordingly.

In this study, we describe our community’s use of human-centered design (HCD) methodology to identify opportunities for redesigning pregnancy care, with the goal of reducing racial and socioeconomic disparities faced by Medicaid-insured individuals. Through a community-health system partnered process, we sought to identify important insights and opportunities to guide redesign efforts and to develop concrete and desirable prototypes for implementation.

## Methods

### Study design and setting

Between November 2017 and October 2018, we conducted a prospective observational study using HCD methodology to identify and develop community-linked, system-level solutions to address the needs of Medicaid-insured pregnant people in San Francisco, California. Foundational to this work was the recognition that pregnant people living on low incomes receive care and support in multiple settings outside of their prenatal providers’ office, including government agencies (such as Medicaid enrollment offices), public health programs (such as the Women, Infant and Children’s program) and community-based organizations (CBOs); our process focused on understanding the entire pregnancy care experience from the perspective of the pregnant person, and to identify gaps and opportunities within and between settings. This work was a collaboration between the San Francisco Respect Initiative, housed within the Department of Obstetrics, Gynecology and Reproductive Sciences at the University of California, San Francisco (UCSF), and The Better Lab, a mixed-methods research center at San Francisco General Hospital (SFGH). The study protocol was approved by the Institutional Review Board of UCSF. Informed consent was obtained from all participants who took part in an in-depth interview or focus group discussion.

### Human-centered design

**Fig. 1:**
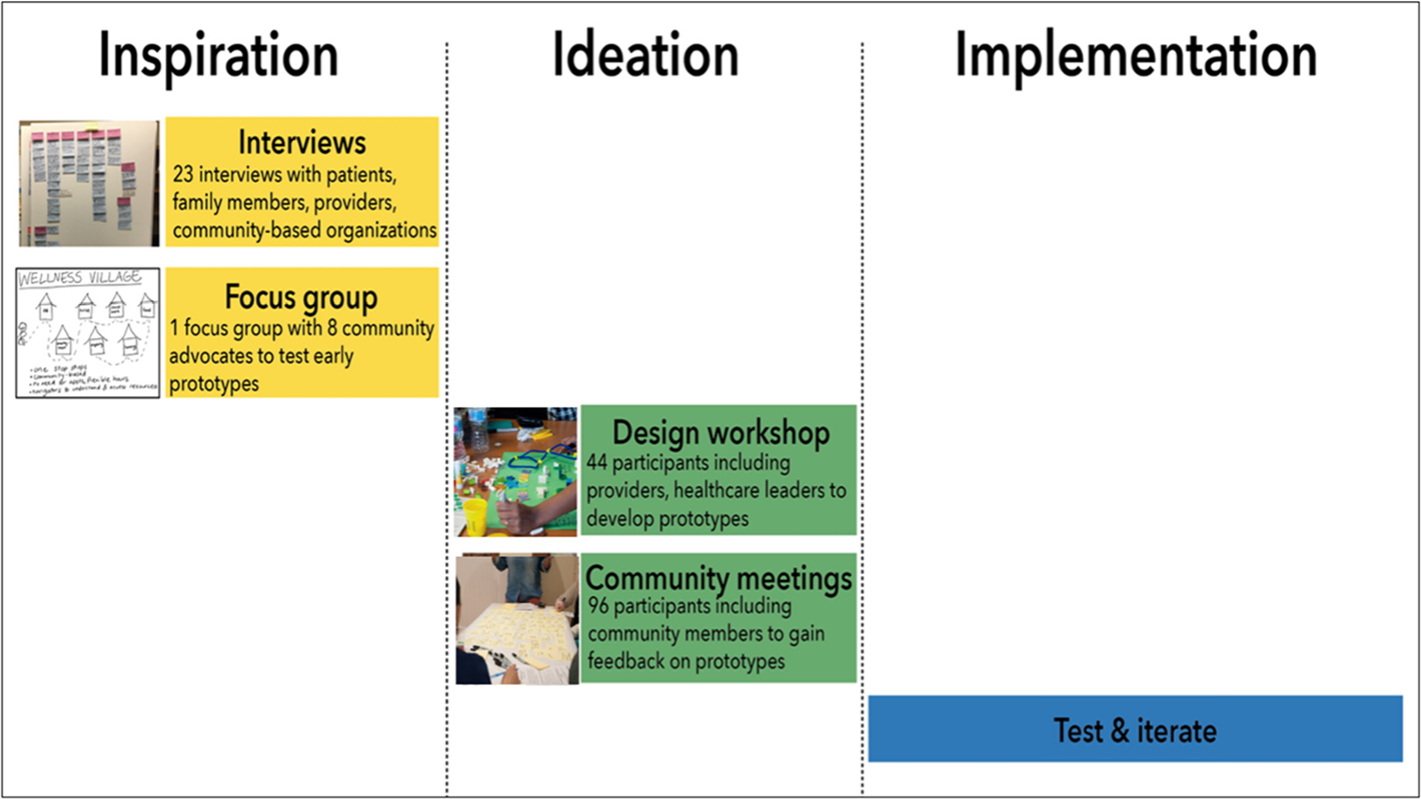
Activities that comprise the Inspiration, Ideation and Implementation phases of the Human-Centered Design process. During the Inspiration and Ideation phases described in this study, insights and themes from target users are made into actionable opportunities, as depicted in Fig. 2

HCD uses ethnographic research to understand the values and needs of stakeholders, and a structured and iterative process to develop innovative solutions that prioritize diverse stakeholders’ needs and preferences. The use of HCD methodology in healthcare has been growing over the past decade, with evidence of improved healthcare access and outcomes [14–16]. HCD involves participatory design, or co-design, where healthcare users (patients) are engaged early in the process as partners in idea generation, which contextualizes and incorporates their values into the final outcome. This approach disrupts traditional processes in which researchers, healthcare providers, and administrators design new models of care based on research studies and expert opinion, and allows the perspectives of healthcare users to be integrated into all stages of the process [17]. As such, HCD was an optimal approach given our goal to identify unmet needs and center the experiences of marginalized groups who are disproportionately represented in the Medicaid-insured perinatal populations – and then to co-design effective solutions. Here, we describe the first and second phases of our HCD process: ‘Inspiration’ and ‘Ideation’ (Fig. 1) [18]. The ‘Inspiration’ phase included qualitative research through individual semi-structured interviews and a focus group with key stakeholders. Qualitative data from these interviews were synthesized to identify themes and inform the creation of insight statements and opportunities. The ‘Ideation’ phase included brainstorming and prototyping solutions, with feedback from users and relevant stakeholders. The third phase, ‘Implementation’, is outlined but will be further described and evaluated in future studies.

### The San Francisco Respect Initiative Advisory Group

Before our study began, the San Francisco Respect Initiative assembled a diverse advisory group of 14 individuals to ensure multi-stakeholder participation across all aspects of the HCD process, and accountability around future implementation of the resulting prototypes (Table 1). Community members were recruited from an advisory board assembled by the California Preterm Birth Initiative, comprised of mothers with lived experience of preterm births, frontline community health and social service providers, and representatives of community-based organizations, and were from racial identity groups historically excluded from research and decision-making processes. Half (7/14) of the advisory group identified as Black, 21% (3/14) as Latinx, 14% (2/14) as White, and 14% (2/14) as Asian; all group members identified as women. The advisory group met monthly and was co-led by the project leader and one of the community members. Prior to starting the HCD process, the advisory group collaboratively articulated guiding principles for the work, including “Community should feel this is their solution”, “ … that their [community members’] voices are valuable,” and “ … that they [community members] had ownership in the process and outcome.” Once the HCD work began, the core research team met weekly and included the project leader (MN), the community member co-lead (SW), and design researchers from The Better Lab (AS, LC, DP, JL).

**Table 1:** Multidisciplinary Advisory Group Members

**Community Members (4)**	
Four Black and Latinx identifying SF residents	
**Health system workers (4)**	
Midwife – clinician and administrator	
Midwife researcher	
Women’s Health Clinic medical assistant - SFGH	
OB/GYN resident physician	
**System Leaders (4) from SF Department of Public Health or UCSF**	
UCSF Center of Excellence in Women’s Health	
UCSF Child Health Equity Initiative	
SFDPH – Perinatal Service Coordinator	
SFDPH – Black Infant Health Program	
**Project Leader**	
Generalist –OB/GYN physician - SFGH and UCSF	
**Facilitator**	
Expert in community engagement and equity driven initiatives (UCSF)	

*Abbreviations*: *SFGH* San Francisco General Hospital, *SFDPH* San Francisco Department of Public Health, *UCSF* University of California San Francisco

### Inspiration phase: Qualitative Data Collection & Analysis

#### Interviews and focus group

Purposeful sampling was used to recruit stakeholders that represent a broad socio-demographic cohort with diverse pregnancy care experiences. The goal was to achieve maximum variation to document unique and diverse variations in how people have experienced pregnancy care [19]. Eligibility criteria for pregnant persons and partners who were interviewed included the following: 18 years old or older; experience of being Medicaid-insured; living in San Francisco; preparing to become pregnant, currently pregnant, or recently pregnant; or being a partner of an individual who was currently or recently pregnant. Individuals were recruited through provider and staff referrals at the SFGH women’s health clinic and at local community organizations that support pregnant people, primarily through referrals and flyers posted at these sites. Health professionals, providers, community health workers, community members, and activists chosen for interviews were recruited through referrals from our advisory group and word of mouth. All interviews were 1 h long and performed in a location of the interview subjects’ choice, including but not limited to community health centers, places of work, and The Better Lab offices. Participation was voluntary and participants were compensated $50 for their time.

Interviews were semi-structured and included open ended questions about the experience of being pregnant, and/or providing or receiving pregnancy care in San Francisco (Additional file 1: Appendix 1). Interviews were conducted by 2–4 members from the core research team. Each interview was led by one design researcher, while the rest of the team recorded notes and asked follow-up questions. To maintain participant confidentiality all interviews were anonymous and were not audio-recorded. One focus group was conducted with a group of 8 community advocates who were part of an existing group assembled by UCSF’s California Preterm Birth Initiative to work with researchers on developing and implementing interventions aimed at preventing preterm birth. The focus group was presented with four early prototypes, to elicit feedback and discussion that would inform further prototype design [20]. These early prototypes emerged from analysis of the first eleven interviews which uncovered some recurrent themes and challenges; and represented early concepts such as a “wellness village” that would house all resources in one location, and a “doula-run clinic,” where a non-clinical, peer support worker was the central member of the care team. Responses from the focus group were anonymous and not audio-recorded to maintain participant confidentiality. One design researcher took detailed notes during the focus group to capture opinions and feedback. Notes from each interview were cross-compared between notetakers to ensure accurate depiction of the interview, removing any discrepancies and biases as they arose.

#### Data analysis

**Fig. 2:**
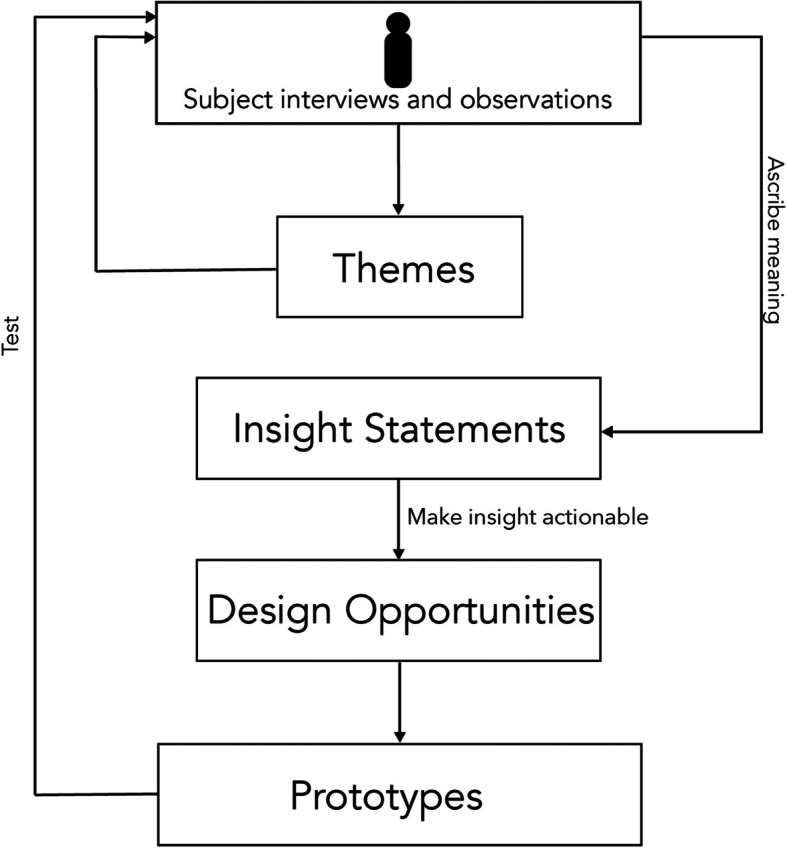
The inductive analysis process starts with qualitative data from users, which is then distilled into themes, contextualized to form insights, and translated into actionable opportunities. These design opportunities are then translated in prototypes that can be implemented and tested in a real-world context

Data analysis followed three steps in accordance with the HCD methodology: 1) Identification of key themes; 2) Development of “insight statements” based on the themes; 3) Translation of each insight statement into “design opportunities” (Fig. 2) [18].

#### Themes

Qualitative data were analyzed using a general inductive thematic analysis approach until thematic saturation was achieved [21–23]. More specifically, each set of notes was reviewed to independently extract quotes or ideas that represented discrete themes. The researchers then collaboratively discussed the themes until consensus was reached about the primary ideas/perspectives shared within each interview. The core research team then discussed all interviews to agree on overall common themes across interviews and identify exemplar quotes to represent each theme. Themes were finally reviewed by the advisory group to ensure the completeness and accuracy of the data from the community and other advisors’ perspectives.

#### Insight statements

The core research team reviewed the themes and developed insight statements. An insight statement is a short sentence that represents user perspectives, motivations, and tensions from the thematic data to define a human need [24]. This approach is specific to HCD, and novel to the academic literature for qualitative data analysis [18]. The goal of developing insight statements is to ascribe meaning to thematic data [25]. The insight statements were reviewed by all members of the core research team and advisory group, discussed and refined until consensus was reached.

#### Design opportunities

Insight statements were then translated into design opportunities. Design opportunities propose actionable ways to address the challenges described in the insight statement and inform the development of low-fidelity prototypes that can be tested with users in subsequent phases of design research [25]. As with insight statements, a list of design opportunities was initially developed by the core research team and then refined through discussion with the advisory group.

### Ideation phase: brainstorming, prototype development and testing

#### Brainstorming: design workshop

To start the ideation phase, a diverse group of healthcare stakeholders were assembled for a four-hour design workshop. A list of potential stakeholders was created by members of the advisory group to ensure representation from different roles involved with perinatal care of Medicaid-insured SF residents, including clinicians with different roles, licensure, and sites of practice (SFGH and UCSF); decision-makers from the major healthcare delivery systems and the Department of Public Health; payers; researchers; leaders from community-based organizations (CBOs); and community members. Stakeholders were invited to attend via direct email from advisory group members.

Insights, supporting themes and quotes were presented to workshop attendees in a structured format using Microsoft PowerPoint slides (Microsoft Corporation, Redmond, USA). After insights were presented, participants were given the opportunity to vote on 6 insights: selecting 3 insights around challenges that they would like to address soon, and 3 around challenges to address in the future. After a group discussion, participants were organized into four groups. Each group chose a brainstorming prompt and its associated insight statement. These brainstorming prompts are known as ‘How Might We’ (HMW) questions, that are written to enable stakeholders to generate solutions to the insight they chose to address. Once the brainstorming was complete, the discrete ideas were organized into categories and stakeholders developed early prototypes to represent these categories.

The core research team and advisory group met several times after the design workshop to review all insights generated from the interviews and focus group, and all opportunities, discrete ideas, and early prototypes generated from the workshop. The focus of these meetings was to consider the outputs of the workshop, alongside data from the interviews and focus groups, to identify a set of initial prototypes that would encapsulate all *design opportunities* identified in the inspiration phase (Table 3). The advisory group’s Guiding Principles were used to ensure that the solutions put forward encapsulated the expressed needs of community members. This process resulted in identification and description of the initial prototypes (Table 5) to be presented for community feedback and scoring.

#### Prototyping: community meetings

Stakeholder feedback and refinement of prototypes occurred through a series of community meetings, focused on obtaining feedback from residents living on low-income in San Francisco. These meetings included a “community design fair,” followed by 3 additional community gatherings held at partnering organizations. The community design fair was organized by the core research team and held at a community center, with advertising done through flyers distributed at clinics, community organizations, and direct outreach. The subsequent community gatherings occurred at existing events or meetings (i.e., annual health fair and support group) that were being held at community organizations serving either the Black community or pregnant people living on low incomes in SF. Members of the advisory group approached the organizational leaders to request 30–45 min of time during these events to obtain feedback on prototypes from attendees. Advertising and outreach was done by each host organization.

At each of the four events, prototypes were presented and then feedback was elicited from attendees. For each prototype, a team member gave a 30–60 s verbal description and some prompt questions (Additional file 1: Appendix 2). Participants were then given up to 5 min to provide feedback on a questionnaire (Additional file 1: Appendix 3), including a rating of each prototype on a 5-point scale based on “how much (they) like(d) the idea,” responses to specific questions, and any other feedback. The initial questionnaire contained 22 structured items and was modified to 13 items as the prototypes were further iterated and prioritized.

During the design fair and one other community meeting, written feedback was followed by a 2-min brainstorming session led by a team member in groups of 8–10 participants. Ideas that emerged from these sessions were captured by a team member on post-it notes, which were then grouped according to common themes and photographed for record-taking.

The advisory group and core research team used rating data, as well as written feedback from the community meetings, to identify the prototype features that were liked most and to iterate each design to incorporate important features. The advisory group then finalized prototypes for implementation based on the scoring and feedback from community meetings, discussion about levels of effort and impact, and a commitment to including those interventions considered to be “low” effort and known to be feasible, as well as those considered “high” effort with less certain feasibility, but potential to create the most transformative impact.

## Results

### Design process participants

Table 2 summarizes the participants involved in each phase and activity. A total of 31 participants engaged in the ‘Inspiration’ phase, including 23 individual interviews with stakeholders and one focus group with 8 participants. Pregnant or previously pregnant participants – although all covered on Medicaid – had received care in different outpatient centers and birthing hospitals, thus representing a range of experiences. In total 19/31 (61%) participants were community affiliated (e.g. patients/pregnant persons and families/caregivers) and 39% were working within the public health or healthcare delivery institutions. Of these participants 39% identified as Black, 13% as Latinx, 19% as Asian, and 29% as White.

**Table 2:** Design phases, activities, outcomes participants and outcomes

PHASE:	INSPIRATION	IDEATION –Brainstorming & early prototyping	IDEATION- Prototype refinement
**Activity:**	• Interviews • Focus Groups	• Design Workshop	• Community Design Fair • Presentations at 3 groups assembled by local CBOs • Advisory group discussions
**Participants:**	• 31 participants	• 44 participants	• 96 participants • Advisory group members
**Participant characteristics**	• Pregnant persons/partners (*n* = 8) • Community representatives (*n* = 11^a^) • Clinical providers / researchers (*n* = 12)	• Care delivery clinicians/ providers/leaders (*n* = 22) • City program staff (DPH/HSA)/leaders (*n* = 4) • Medicaid health plan staff/leaders (*n* = 3) • CBO partners and Community members (*n* = 6) • Researchers (*n* = 5) • Designers (*n* = 4)	• Community and other stakeholder participants at gatherings held at CBO community events and meetings (*n* = 96): • 88% female • 68% reproductive age (< 45 years old), • 86% non-white • 78% ever pregnant • 64% receiving/received care on Medicaid
**Outcomes:**	• 7 insights • 7 opportunities	• 162 discrete ideas • 8 prototypes	• 3 overarching perinatal redesign opportunities • 4 prototypes for implementation

*CBO* Community Based Organization, *DPH* Department of Public Health, *HSA* Human Services Agency

^a^ 8 community advocates participated in group discussion rather than one-on-one interviews

A total of 140 participants were involved in the ‘Ideation’ phase (brainstorming and prototyping), including 44 participants in the design workshop and 96 participants at the community design fair and subsequent community meetings. The design workshop focused on a broad range of healthcare stakeholders, whereas the community design fair and subsequent community meetings were more focused on community members (Table 2).

### Inspiration: results of Qualitative Data Collection & Analysis

Analysis of interviews and the focus group revealed seven insights that represented unique challenges, tensions, and perspectives of perinatal individuals and their providers. Table 3 describes each insight, supporting quotations and associated design opportunities.

**Table 3:** Key insights, supporting quotes and opportunities from the Inspiration Phase

Insight	Themes & Supporting Quotes	Design Opportunities
1. Marginalized people are not welcomed as equal, trusted partners in their care	**Unequal power dynamics breed lack of trust** “My care was ‘sporadically informative’. They fed me information only when they wanted. I got only information when I pressed for it … I always felt they knew something that I didn’t.” - Pregnant person **Providers make (often incorrect) assumptions about what pregnant persons want to know** “I just tell the mom that the baby is perfect. Because the value of reassurance to the woman is so much greater …”– Obstetrician **Experience with racist stereotypes can make pregnant persons scared to advocate for themselves** “[Pregnant persons] often times are just seen as the angry black women who are vocal and argumentative … they learn to be quiet.” – Midwife	(a) (Identify ways to) proactively shift power dynamics between pregnant persons and their providers to foster trust and partnership (b) Help healthcare team members to recognize and undo their own biases
2. Every touchpoint is essential, and one bad interaction can change the course of care	**Disrespect is communicated in many different ways, and adds up** “Clinic is busy, but does that mean you can’t give eye contact? Does that mean you leave [the pregnant person] in the hallway?” - Pregnant person “Something like being scheduled for the wrong time in your clinic appointment, little things that nobody likes. However, in the context of someone who’s lived a life where they’ve been a victim of the spectrum of racism, those things add up in a big way.” – Community Activist **If pregnant persons can’t trust that they’ll be treated well, they’re less likely to engage** “If I feel as though I’m not worth your time, I’m not gonna come back.” - Pregnant person **Lack of trust is dangerous** “There was a patient from Haiti and this was her 2nd baby. She had a routine c-section. An hour after surgery, she arrested, & they couldn’t bring her back. Later found out that someone had told her not to get pregnant again. She intentionally didn’t tell anyone about that. To me, there wasn’t trust there somehow for her to disclose that.” –Family Medicine provider	(a) Approach every interaction as an opportunity to earn trust
3. The system stigmatizes lived experiences, and then requires people to re-tell their stories multiple times	**History stigmatizes one’s care** “People see mental health problems and they just stiff arm them.” - Pregnant person **Repeating a traumatic history breeds shame.** “A lot of people feel that they were being judged for their story. The more they told their story, the more chances they have of being judged.”– Midwife	(a) Create less burdensome mechanisms for pregnant persons to communicate their stories across care transitions
4. Racism affects how people show up, and then negatively impacts their care	**The burden of structural and interpersonal racism impacts how people show up** “It is the hardest thing for me when a woman comes in and so much has already happened to them that the option to rapport build is just not there at all” – Midwife “Women who are vulnerable don’t feel like they can speak.” –Midwife **The system punishes pregnant persons who are impacted by care barriers caused by systemic racism** “At [care institution], I was turned away if I was late.” –Community advocate	(a) Structure each visit around what the individual says they need that day, and create mechanisms for them to easily communicate this with providers
5. Barriers to care are significant	**Being “compliant” with care isn’t as easy as it sounds** “For someone who’s at risk for hypertension and pre-eclampsia who needs to have her blood pressure checked, no one thinks to ask what that involves – childcare for three kids, buses, time off work. We [providers] just say cavalierly that they need to get their blood pressure checked and not think about maybe teaching them about how to take their blood pressure, the implications this may have on their lives and what it means for their lives.” -Midwife **It’s hard to focus on pregnancy when you’re focusing on survival.** “I didn’t know the due date, but I knew I’d get 3 days with a hot shower.”– Homeless Pregnant person **Those who need the most resources have the least around them** In Noe Valley there is all kinds of stuff; I don’t want to have to take a bus just because I don’t have that in my community. -- Pregnant person living in Public Housing (Potrero Hill)	(a) Make care and services more valuable and easier to access, especially for those who face the worst outcomes and the most barriers to care
6. Lived experience (social, medical, or cultural) makes pregnant persons “experienced” and in a position to help others	**History makes the expert with lessons to share.** “[Women with lived experience:] They’re experts, consultants, partners.” – Community Health Worker **Pregnancy is a vulnerable time, and having support is essential** “To have other camaraderie with women who are in the same situations as you are, to see a light at the end of the tunnel. It’s hopeful, inspiring…nice.” - Pregnant person “I feel like coming into motherhood I’m not equipped, not adequate. I’m going to mess her up.” -Pregnant person	(a) Incorporate people with lived experience as valued members of the healthcare team who can help others
7. Pregnancy is treated like a disease, rather than a life-changing event for pregnant people and their families	**Over-focus on the pregnancy and not the pregnant person is de-humanizing.** “In the process I got weak. I got lost. Because no one cared for *me*.” --Pregnant person **Pregnant people want the celebration, the proverbial baby shower.** “You want everyone more excited than you are....It’s supposed to be the best time of your life –I didn’t have that opportunity.” – Homeless Pregnant person **Community is as important as medical care for healthy outcomes** “Ultimately for me, it’s support and community. We can get a lot of things from doctors, we can get information up the wazoo. But it is supporting what we believe and what we want for the future of our child that is important to me.”	(a) Use resources to provide more community support, rather than more medical care, during pregnancy

Insights broadly fell into two categories. The first category described the role that racism and discrimination—on interpersonal, institutional and systemic levels—plays in creating major barriers to the access, experience and value of pregnancy care interactions. Participant experiences highlighted that care is fraught with unequal and uncomfortable power dynamics between patients/pregnant persons and clinicians (Insight 1), often feels disrespectful and judgmental (Insights 2, 3) and does not acknowledge the significant systemic barriers that impact one’s ability to access and comply with care (Insight 4, 5). These insights highlighted the need to pursue opportunities that would improve access to care, make care interactions with clinic providers and staff more positive, and allow members of the healthcare delivery system to earn back the trust of communities that have been historically marginalized and harmed by medical care.

The second category comprised of the over-medicalization and non-inclusive nature of pregnancy care. Insights demonstrated that pregnant people want to feel celebrated, rather than pathologized, during this life transition (Insight 7), value the support and wisdom of their peers (Insight 6) and want to be informed and equal partners in care decisions (Insight 1). These insights highlighted the importance of findings opportunities to shift the care experience away from one where pregnancy is approached as a medical problem, and towards one where pregnancy is approached as a life transition that requires as much focus on the social, emotional, and practical aspects as on the medical and clinical aspects.

### Ideation: results of brainstorming, prototyping and refinement

Brainstorming at the design workshop, in response to the insights and opportunities presented, generated a total of 39 HMW statements and 162 discrete ideas. As an example, one HMW question that emerged from the opportunity “Approach every interaction as an opportunity to earn trust” was “How might we embed empathy into every aspect of care?” This subsequently led to 51 discrete ideas including “Let women say what they need, then provide that!”, “Build a village model of care,” and “Bring the resources to them.” Table 4 lists selected other examples of HMW statements and ideas generated from them.

**Table 4:** Selection of “How Might We…?” (HMW) questions and associated ideas from the design workshop

Question	Selected Ideas
HMW acknowledge and address interpersonal and systemic racism in our system of care and support?	• Peer advocates to help empower women • More diversity in hiring and teaching • Listen to me with empathy
HMW help women be the experts in telling their own story?	• Pregnant persons interview their provider • The MD is the consultant
HMW help providers and pregnant persons have transformative experiences?	• Recognize women’s expertise [by asking]: “tell me your story” • Pregnant person decides next visit agenda
HMW we ensure our pregnant persons’ priorities are our priorities?	• Meet them where they are ➔ mobile • One stop shop ➔ wellness village

Table 5 describes the 8 initial prototypes that were presented at the community design fair and were iterated on in response to scoring and feedback throughout the subsequent community meetings. Overall scores from all quantitative feedback are listed, along with a summary of feedback and discussion that led to the listed conclusion about implementation.

**Table 5:** Initial Prototypes, participant scoring and feedback, conclusions and rationale

Prototype	Brief Description and design opportunity addressed	OVERALL SCORE	CONCLUSION	Summary of participant feedback and Advisory Group conclusions
SUPPORT SISTER	Person on your care team who has gone through this experience, and is there to guide, support and get you what you need throughout your pregnancy and after. Design Opportunity 1(a), 3(a) 6(a), 7(a)	4.6 (out of 5)	Adopted for implementation	**Summary:** Many comments revealing high enthusiasm for this concept **Refinement:** Questions emerged around if Support Sisters should be hired within health systems or be community based, recognizing that within health system allows for important integration with health system, but may compromise ability of support sister to effectively advocate for client if in conflict with health system staff. **Rationale and final prototype details:** • High impact for providing support, helping navigate resources, and identifying/mitigating interpersonal racism • Prototype to specifically focus on how community-based Support Sisters can be sustainably integrated into healthcare teams to allow for care that is more comprehensive (provides practical, emotional, and social support), is well-coordinated (insight and outside of healthcare system), and provides cultural sensitivity and lived experience.
COMMUNITY CENTER FOR PREGNANCY AND YOUNG FAMILIES	A community center as a “one-stop-shop” that provides clinical and non-clinical services, and support for pregnant people and young families Design Opportunity 2(a), 4(a), 5(a), 7(a)	4.6 (out of 5)	Adopted for implementation with refinement	**Summary:** Many comments revealing high enthusiasm for this concept **Refinement:** Comments revealed that people thought this would be most helpful if within one’s own neighborhood. Broad array of services and goods desired. **Rationale and final prototype details**: • High impact for reducing barriers to care, tackling systemic and institutional racism, shifting power dynamics, and investing in community support. • Recognizing that this could not exist in every neighborhood, this prototype was combined with *“Services that come to you”* and *“Build community with your care team”* prototypes (below) to create the “Pregnancy Village” prototype, as described in results section. Pregnancy Village brings services and goods into neighborhoods making them easier to access, while also providing an environment to foster community support, shift problematic power dynamics with providers, and develop more trusting partnerships between pregnant people and service providers.
SERVICES THAT COME TO YOU	A mobile unit that travels to your neighborhood with helpful services and offerings. Design Opportunity 2(a), 5(a)	4.6 (out of 5)	Adopted for implementation with refinement	**Summary:** High enthusiasm for services being brought to one’s own neighborhood Concerns were around how to make this look and feel respectful—i.e. would people be lining up waiting for services? How would the mobile unit look and feel inside – “cold and clinical” versus “warm and comfortable”? **Rationale and final prototype details:** • Combined with *Community Center* prototype (see above) to create “Pregnancy Village” prototype, as described in the results section of manuscript. • Mobile unit a necessary part of Pregnancy Village prototype to deliver more private (clinical) services
PRENATAL CARE FROM HOME	Video chat with your pregnancy care team from home, instead of coming into clinic. Design Opportunity 5(a)	4.1 (out of 5)	Adopted for implementation	**Summary:** Feedback was mixed, with some participants enthusiastic about the convenience of this option and others not sure it would be personal enough. **Refinement:** Must be implemented in an equitable way to ensure access and value for those with low resources and varying levels of digital access and literacy. **Rationale and final prototype details:** • Low-effort, high-impact intervention for pregnant persons who would want this option (and would not negatively impact those who wouldn’t). • Should be implemented as an option across the safety-net system so is an option, when clinically appropriate, for any pregnant person who would like it. Should not be required just because eligible.
USEFUL TRANSPORTATION	Provide transportation options that offer pregnant person education and will check you in to clinic on the ride. Design Opportunity 5(a)	4.5 (out of 5)	Not adopted for implementation	**Summary:** High average score but not many comments, revealing low enthusiasm. **Rationale:** Given pregnant persons get care from multiple different clinics, this would be a high-effort intervention with unclear impact. Additionally, based on enthusiasm for bringing clinical services into neighborhoods, decided to prioritize “Pregnancy Village” model over this prototype.
BUILDING COMMUNITY WITH YOUR CARE TEAM	Activities that allow you to get to know your doctors and midwives in a setting outside the clinic, to build trust and relationships. Design Opportunity 1(a), 1(b), 2(a) 7(a)	4.1 (out of 5)	Not adopted for implementation	**Summary:** Some enthusiasm, but comments revealed that other mechanisms such as continuity of care and longer appointments were most important factors in building trust and relationships. **Rationale:** Included this prototype/concept into the “Pregnancy Village” prototype as described in the results section.
CHOOSE FEWER VISITS	Reduce minimum number of doctor or midwife visits to five and have other visits with whoever you choose from care team (for example, support sister or a healthcare educator) Design Opportunity 6(a) 7(a)	3.83 (out of 5)	Not adopted for implementation	**Summary:** Poor enthusiasm for this concept, with many people concerned about the safety of having so few visits with providers, and wondering how they would know when they need to see a provider **Rationale:** While studies show five clinical visits safe for low-risk pregnant people, pregnant persons are not comfortable with this approach.
LEARN THROUGH EXPERIENCE	Learn about pregnancy-related topics through the eyes of a peer who has experienced it using virtual reality technology. Design Opportunity 7(a)	3.3 (out of 5)	Not adopted for implementation	**Summary:** Poor enthusiasm for this concept, though did think might be a helpful adjunct to different classes/ education that are already available (lactation, labor & delivery, birthing) **Rationale:** Low impact

Three finalized prototypes were selected by the advisory group to move forward for the final phase of the HCD process: implementation.

1. **Universal access to a “Support Sister” (community doula or perinatal health worker) for all Medicaid-insured pregnant people**

Several key features emerged around how to make the Support Sister most impactful: establishment of the relationship early in pregnancy and ensuring its continued support beyond the traditional postpartum period, someone who is easily accessible, and someone who is available to help navigate the system and the pregnant person’s needs. The need to be well-connected to the healthcare system was an important feature, so the Support Sister could help overcome barriers within the health system, like helping get an appointment and helping advocate if there is difficult communication with staff and/or providers. Other desired characteristics of this peer support person included being from one’s own community; helping to navigate services outside of the clinic; being reliable, empathetic, familial, and able to speak from experience; using media to help address basic concerns and share knowledge; and being readily available.

2. **Telemedicine (video visits) as an alternative option to in-person visits**

While many people felt that they would prefer to see their provider in person, others appreciated the option for video visits because of their ease of attendance. Participants felt this would be particularly helpful for certain types of appointments such as receiving test results and one-time consultations for counseling. While this prototype had an overall lower score than some others, comments from feedback suggested that having a remote option for care could provide considerable benefit to those whose care access is limited because of significant practical barriers to care. Given that telemedicine implementation was considered “low-effort,” the advisory group determined that this was an important prototype to purse to reduce disparities in care access.

3. **The “Pregnancy Village” model to deliver care and services into neighborhoods through a one-stop-shop**

The importance of delivering care and services in a way that is less burdensome, and that better responds to the needs of communities, was clear. The Pregnancy Village prototype was designed to achieve this goal - delivering multiple pregnancy-related services, such as clinical care, public entitlements (e.g. Women, Infant and Children’s program benefits, Medicaid enrollment), and wraparound services (e.g. breastfeeding support, community-based services) into high-need neighborhoods, at one place and time, on a recurring basis. The prototype design also recognizes the value of creating a new *environment* for care delivery: one that is be free from the historical trauma and harm that have been experienced by communities of color in healthcare institutions, would shift away from the power dynamics that people feel when interacting with providers inside of institutions, and would provide a safe, healing, and positive space focusing on community wellness.

## Discussion

Socioeconomic and racial inequities in perinatal care access, experience, and outcomes exist across the United States [26, 27]. The approach to tackling these inequities in any given community will depend on the local context of care delivery and policy, resource availability, and other social and community inequities. Our work describes the process of using HCD methodology to first understand pregnancy care experiences from different stakeholders’ perspectives, and to then use this understanding to design promising interventions. With the goal of this work being to identify feasible and sustainable interventions, we included health system stakeholders (i.e. front-line providers and administrative decision-makers) throughout the process, so as to understand diverse perspectives that would impact implementation. However, we explicitly focused our process on centering the experiences of individuals receiving care (particularly those groups that have been marginalized within healthcare settings and other systems), and designed solutions in response to these experiences. Our HCD process revealed both short-term opportunities (use of technology and community-based care delivery models) and long-term investments (understanding and responding to the needs of marginalized communities) to improve perinatal care access, experience and outcomes of Medicaid-insured pregnant people in our urban community.

Findings from our ‘Inspiration’ phase are consistent with existing literature that demonstrates why the traditional U.S. system of pregnancy care delivery results in poor access, experience, and outcomes among low-income people, and especially low-income people of color. The barriers to care access place significant and disproportionate burden on those who must take unpaid leave to attend appointments, have unreliable transportation, and face other practical constraints [8]. Discriminatory and racist practice patterns are commonplace, making it extremely challenging to feel safe and trust the care provided [28]. Additionally, pregnancy care is overmedicalized with not enough investment and focus on the non-medical support needed for people to thrive, such as peer support through doulas and community health workers [29]. Our work contributes further by describing the use of HCD methodology to translate these insights into concrete solutions that could be prototyped for local community ratings and feedback. The opportunities and prototypes include changes that could be made at each level of the healthcare delivery system: individual (e.g. approach every interaction as an opportunity to earn trust), institutional (e.g. deliver care and services into neighborhoods), and systemic (e.g. payment and practice policies that support integration of community health workers and doulas into care).

The use of HCD methodology in healthcare is becoming more common, with a focus on centering the user experience when designing new interventions and care models. Traditional qualitative research is able to describe the barriers and facilitators to high-quality care and to test interventions, but often misses the step of co-creating solutions that are relevant and preferred by the local community of interest [30]. HCD allows for such co-creation and is particularly valuable when working within a local context--utilizing local expertise to identify problems and opportunities and uncover promising solutions [31]. In other words, our application of HCD is an explicitly action-oriented endeavor, rather than a design thinking process alone, which aligns with previous work in this space. In perinatal care, HCD has been used to develop specific healthcare interventions or programs for defined populations within a care delivery system, such as adolescents, people living with substance-use disorder, and low risk people who may need less in-person care than the traditional model dictates [32–34]. In our study, we sought to better understand the entire pregnancy journey of Medicaid-insured individuals within our community, inside and outside of the care provided within clinical care sites, to build on this literature. Finally, it is important to note that the most promising, novel perinatal care models – such as group prenatal care, home visiting programs (Nurse Family Partnership®) and free doula care – are already available to residents of color living on low incomes in San Francisco [12, 13, 35], yet our city’s significant inequities in perinatal care and outcomes persist [36]. Therefore, we intentionally used HCD within this study at the broadest level possible, so that solutions would not be confined to a specific part of the system (e.g. within one clinic, hospital, city/public health agencies, or CBO), and could include those that exist *between* parts of the system (e.g. opportunities for better care coordination).

We focused heavily on engaging leaders from across organizations and sectors starting from early in our HCD process. Successful implementation of the prototypes we developed would ultimately depend on buy-in from organizational leaders, and it was critical that they gain understanding of the problem before being asked to implement solutions [37]. We used various approaches to engage with organizational leaders. The project leader spent significant time meeting with them one-on-one to introduce the background and plans for the HCD process. Organizational leaders were asked to appoint a representative to our advisory group, adding to the group’s collective sphere of influence. Finally, we invited multi-sector leaders to the design workshop in which insights were shared and early prototypes were built. These leaders included decision-makers from the safety-net care system, public health department, human service agency, and health plans, among others. This process required a significant amount of time over the course of 1 year but was considered essential to ensure willingness to implement solutions later. Indeed, these efforts were successful in that all three of the finalized prototypes are currently being implemented within our community, with telemedicine (video visits) now a standard offering for pregnant people receiving care within the safety net system and Pregnancy Village and Support Sister integration pilots launching in Summer and Fall of 2021 respectively.

While the HCD ‘Implementation’ phase plans are broadly summarized in this study, rollout and subsequent evaluation of each prototype is ongoing and continues to be iterated in response to user feedback. For our work to make “Support Sisters” universally accessible to Medicaid- insured pregnant people, we are working closely with two perinatally-focused CBOs in our community to better integrate their doulas and perinatal health workers into the broader care team, and to create sustainable models for revenue generation. Implementation of video visits within our safety-net system started in our Maternal-Fetal-Medicine clinics, with iteration of workflows and user-friendly tools ongoing in response to patient and provider feedback. Finally, the *Pregnancy Village* prototype will launch Summer 2021 in San Francisco’s Bayview neighborhood, using a community-partnered process which will incorporate real-time feedback and iteration throughout implementation.

Limitations of our study include the local focus of HCD work, which may make the themes and opportunities less generalizable to other communities. While the outcomes of the HCD process are most valuable when tailored to a local context, the use of purposeful sampling and consistency of our themes with those in the national literature suggest that the opportunities we identified may apply to other communities. An additional limitation to our study is the decision not to audio record and therefore transcribe the interviews, leading to potential bias and incompleteness in the interview data. While audio recordings and direct transcriptions would have ensured data accuracy and validity, we felt it was important to maintain confidentiality and privacy of a population that has experienced significant discrimination and trauma with respect to their healthcare experiences. Finally, our ‘Inspiration’ phase had a modest sample size for qualitative inquiry and our ‘Ideation’ phase included only a few perinatally focused community-based organizations – given the limited number that exist in our city. However, combining their feedback across multiple HCD phases with both open-ended interviews and brainstorming and closed-ended scoring likely reinforced the primary HCD findings in this study.

## Conclusion

Our work supports the strong push for a major overhaul of pregnancy care in the United States. Evidence of low value perinatal care is clear and widespread, with stark and unjustified racial disparities in outcomes and those most vulnerable to these outcomes facing extreme burdens while both accessing and experiencing pregnancy care. The insights, opportunities, and prototypes that emerged from our work may be of use to similar communities seeking to tackle these disparities by changing models of care delivery. For communities eager to take a community-engaged approach to understanding and addressing the specific challenges in their own setting, the description of our HCD work may offer a valuable approach. Given the maternity care crisis in communities across the U.S., intentional and urgent change efforts are critical.

## Supplementary Information

**Additional file 1.** Appendix 1: Interview guide. Appendix 2: Verbal Descriptions and Prompt Questions for Prototypes. Appendix 3: Questionnaires for Feedback and Scoring.

## Data Availability

The datasets used and/or analyzed during the current study are available from the corresponding author on reasonable request.
