# Supplementary material for: Using human centered design to identify opportunities for reducing inequities in perinatal care
Source: BMC Health Serv Res. 2021 Jul 20;21:714. doi: 10.1186/s12913-021-06609-8 (PMC8293556; doi:10.1186/s12913-021-06609-8)
Supplement: Supplementary file 1 — Additional file 1. Appendix 1: Interview guide. Appendix 2: Verbal Descriptions and Prompt Questions for Prototypes. Appendix 3: Questionnaires for Feedback and Scoring. [file 12913_2021_6609_MOESM1_ESM.pdf]

Using Human Centered Design to Identify Opportunities for Reducing Inequities in Perinatal Care : Appendices

Malini A. Nijagal MD MPH,[1] Devika Patel MS,[2] Courtney Lyles PhD,[3] Jennifer Liao MD,[4] Lara Chehab MPH,[2] Schyneida Williams,[1] Amanda Sammann MD MPH[2]

[1] Department of Obstetrics, Gynecology and Reproductive Sciences, UCSF/ZSFG, 1001 Potrero Avenue, Building 5, 6D-9. San Francisco, CA 94110

[2] Department of Surgery, University of California, San Francisco

[3] Center for Vulnerable Populations, University of California, San Francisco at Zuckerberg San Francisco General Hospital

[4]Department of Emergency Medicine, Jefferson University

Correspondence to: Malini A. Nijagal, MD MPH – malini.nijagal@ucsf.edu

Appendix 1: Interview guide

Using Human-Centered Design to Understand Perinatal Care Experiences for Publicly Insured Pregnant People in San Francisco
Semi-structured Interview Guide

We want to gain a better understanding of the experiences of publicly insured pregnant people in San Francisco.

(Semi-structured interviews will focus on perceptions of perinatal care delivery and the experience of receiving, providing or supporting care for publicly insured women in San Francisco. Topics will be approached through the following follow-up questions.)

Patients: Patients will be asked to describe their experience with the perinatal care system in San Francisco. Families who are interviewed will be asked similar themes as they relate to care for their loved one. Questions will be open ended and will follow themes such as:

- Tell us about yourself.
- Tell us about how you found out about your pregnancy. What was that time like for you?
- Tell us about your experience receiving pre-natal care. What was it like for you? What were some challenges in accessing pre-natal care?
- What did you really like about your experience at the hospital where you received care? What did you not like?
- What was your care like during delivery? After delivery?
- What did you not like about your experience receiving care? What were some challenges you faced?
- What were some of the things that you really needed during your pregnancy and after delivery that you didn’t get?
- If you could change one thing about your perinatal care experience, what would it be? What are your hopes and dreams for the future?

Providers: Providers will be asked to describe their experience providing perinatal care in San Francisco. Questions will be open ended and will follow themes such as:

- What is a typical day like for you?
- What do you like most about your job?
- What are some of the most frustrating parts of your job?
- What do you think that women appreciate the most when they seek care?
- What do you think that women need that they aren’t receiving in their care experience?

Administrators: Program directors and managers and any other hospital or clinic staff involved in perinatal care service delivery will be asked to describe their experience with providing services related to perinatal care for women in San Francisco. Questions will be open ended and will follow themes such as:

- Describe the program/service that you work on.

- What are the goals of this program/service?
- What are the barriers to achieving those goals?
- What are some things that would help to make it better?
- What does an ideal day look like for you?

Appendix 2: Verbal Descriptions and Prompt Questions for Prototypes

SUPPORT SISTER

Navigating your life (not just your health and healthcare) during pregnancy can be incredibly complex. Imagine having a specific, trained person who knows you well and you can call, at any time of day, any day of the week, to be there to support and advise you throughout your pregnancy journey. They are a tool and a friend for pregnant women to rely on, as they navigate the complex system to access care and services.

Discussion Questions:

- What type of person would you want? Same neighborhood? Same circumstances? Same race? Same religion or culture?
- What type of services would you need help with?
- How would you want to engage with them? - would you want them to attend appointments with you, meet with you regularly for social visits or something else?
- Would you be interested in being a support sister and if yes, what type of training should this person have?

COMMUNITY CENTER

What if the resources and help you need to navigate your pregnancy care were located in ONE place? We imagine this center to be a “landing spot” where any pregnant person could go to help figure out the best clinic for you and provide you with important resources. This could include enrollment services for Medi-cal, Calworks, WIC, home nursing and other programs, and could also have a prenatal care clinic on site. We also imagine that it would provide community space to support women and young families, including areas for socializing, gardening/cooking, and providing classes to promote wellness such as cooking, career planning, exercise and stress reduction.

Discussion Questions:

- When would you want to go to this center and why?
- When in your pregnancy would you imagine you would use this center?
- Would you travel across the city to go to a center that had everything under one roof?

SERVICES THAT COME TO YOU

What if the resources and care you need came to your neighborhood? A mobile care unit could come near your home and provide access to commonly-needed services. This mobile unit might have people to help you enroll in services such as Medi-Cal, Calworks, or WIC, or have people that help you understand how and where to get services. The mobile unit might also offer education or provide clinical care. These services would come together to one place in your neighborhood, rather than asking you to travel to obtain these services.

Discussion Questions:

- What services would you want this mobile unit to provide?
- How frequently would you want to access this mobile unit?
- How frequently should this van come to your neighborhood? Weekly? Monthly? Daily?

- Where would you want to meet this mobile unit? Near a common intersection? In front of a community center? Near your clinic? Near the grocery store?

PRENATAL CARE FROM HOME

What if you didn't have to come into the clinic in person every time? What if you could do some of your check-ups from home? Imagine you could attend your doctor or midwife visits by video chatting on your phone, computer or tablet. This may require you to do some simple test, such as measure your own blood pressure or check the baby's heartbeat. Would you be willing to do this? Home video chat visits may be a way to access your care team conveniently and without having to worry as much about transportation, child care and more.

Discussion Questions:

- Do you feel comfortable video chatting on your phone, tablet or computer?
- Do you believe you could get the same care as an in person visit on video?
- Would you want to video chat to replace regularly scheduled visits, or would you prefer this to be an additional visit with other members of your care team?
- Who would you want to video chat with?

BUILDING COMMUNITY WITH YOUR CARE TEAM

Would you be interested in participating in regularly scheduled activities with your doctors and midwives? The purpose of this idea is to bring women and healthcare providers together in comfortable and safe community settings so that they can get to know each other and build trust. We'd organize different events all over the city offering different activities for members of your care team to get to know you and your family outside of the clinic.

Discussion Questions:

- What activities would you want to participate in? Meals? Classes? Physical activities such as hiking?
- How often should these gatherings occur? Weekly? Monthly? Every 2-4 months?
- Which care givers would you want to attend?
- Would you go? If yes, why? If no, why not?

USEFUL TRANSPORTATION

For many women, transportation can be a real, inconvenient problem. Current transportation options may be inaccessible, difficult and/or time-consuming. What if we provided free/low-cost transportation, through a shuttle or ride-sharing service like Lyft, and along the way, we educated you about your visit through your smartphone/or through a phone call? Through your ride to the clinic, you would have the chance to learn about your clinic visit, and the important information you may need to walk into your visit well-informed. We would even check you in so that you don't have to stand in line when you arrive. On the ride home, you would be able to review important follow-up information.

Discussion Questions:

- Would you use a hospital or clinic sponsored shuttle to take you from your community to your appointments?

- If you could design your shuttle ride, what would you want to do? Relax? Learn? Socialize?
- What additional things would the shuttle have to offer? Car seats for kids? Bottled water and snacks? Wifi?

CHOOSE FEWER VISITS

Not all of your clinic visits are medically necessary. For low-risk pregnancies, we now know that it's safe to have just 5 visits with your doctor or midwife – with additional visits only when wanted or needed. What if you could choose to have fewer visits to the clinic? Or have just as many clinic visits as now, but you could choose who you want to see depending on what you need and want that day. For many of your concerns, it may be more convenient, efficient and comfortable to access support in a different way, such as through a "support sister", a community center, virtual care or a mobile unit.

Discussion Questions:

- How often do you want to see a doctor or midwife during your pregnancy?
- Would you feel comfortable replacing a doctor or midwife with an appointment with another member of the care team? How about a peer or someone from your community?
- How would you build your pregnancy journey? Who do you see and when? (timeline?)

LEARN THROUGH EXPERIENCE

An important part of the patient experience is education. However, this is usually focused on providing information through brochures or pamphlets. The education often feels impersonal, irrelevant and is so overwhelming that it is left un-read or not well understood. Imagine being able to immerse yourself, by putting on Virtual Reality goggles, to learn from someone else's experience. It would feel like you are inside of a YouTube instructional video. These videos would be used to show you how other women like you have addressed challenges and/or customized their care.

Discussion Questions:

- Do you feel comfortable putting VR goggles on?
- Would you use this educational technology and if yes, where and when would you want to use it?
- What experiences would you want to see? Delivery? Breastfeeding? How other moms manage motherhood?

APPENDIX 3: Questionnaires for Feedback and Scoring

Version 1: 22 Item

First, we'd love to hear from you:

How old are you? _______________

Gender that you identify with (circle one): Male Female Other

Race/ethnicity/culture you most identify with: _______________

Have you ever been on Medi-Cal insurance? Circle one: YES NO

Have you been pregnant before, or been a partner to someone who was pregnant? Circle one: YES NO

If you have been pregnant, were you on Medi-Cal insurance at that time ?
Circle one: YES NO

Neighborhood you live in or consider home: ___________________________

BUILDING COMMUNITY WITH YOUR CARE TEAM

Description

Get to know your doctors and midwives in a setting outside the clinic, to build trust and relationships.

Questions for You

1. How much do you like this idea? Rate it from 1-5:

**1**	**2**	**3**	**4**	**5**
**Don't love it**		**It's okay**		**Love it**

2. What would you want to see at these gatherings?

3. What would help you build trust with your healthcare provider?

Additional Comments:

SUPPORT SISTER

Description

A person working in the clinic who has already gone through this experience, and is there to guide, support and get you what you need throughout your pregnancy.

Questions for You

1. How much do you like this idea? Rate it from 1-5:

**1**	**2**	**3**	**4**	**5**
**Don't love it**		**It's okay**		**Love it**

2. What does a “support sister” look like to you? What characteristics does this person have?

Additional Comments:

LEARN THROUGH EXPERIENCE

Description

Learn about pregnancy-related topics through the eyes of a peer who has experienced it.

Questions for You

1. How much do you like this idea? Rate it from 1-5:

1	2	3	4	5
**Don’t love it**		**It’s okay**		**Love it**

2. What types of experiences would you find useful to learn about through this medium?

3. What do you wish you had known before giving birth for the first time?

Additional Comments:

USEFUL TRANSPORTATION

Description

Provide transportation options that offer patient education and will check you in to clinic on the ride.

Questions for You

1. How much do you like this idea? Rate it from 1-5:

1	2	3	4	5
**Don’t love it**		**It’s okay**		**Love it**

2. Do transportation issues prevent you from getting to clinic appointments?

1	2	3	4	5
**Never**		**Sometimes**		**Often**

Additional Comments:

PRENATAL CARE FROM HOME

Description

Video chat with your pregnancy care team, instead of coming into clinic.

Questions for You

1. How much do you like this idea? Rate it from 1-5:

**1**	**2**	**3**	**4**	**5**
**Don't love it**		**It's okay**		**Love it**

2. Do you video chat with your friends and family already? Circle one:

YES	NO

Additional Comments:

CHOOSE FEWER VISITS

Description

Reduce minimum number of doctor/midwife visits to 5 and allow you to choose which other team members you want to see (for example, support sister or a healthcare educator)

Questions for You

1. How much do you like this idea? Rate it from 1-5:

**1**	**2**	**3**	**4**	**5**
**Don't love it**		**It's okay**		**Love it**

2. How do you feel about the option of going to a doctor and/or midwife less often during pregnancy?

Additional Comments:

SERVICES THAT COME TO YOU

Description

A mobile unit that travels to your neighborhood with helpful services and offerings.

Questions for You

1. How much do you like this idea? Rate it from 1-5:

1	2	3	4	5
**Don't love it**		**It's okay**		**Love it**

2. Rank these services from 1 (most important) to 8 (least important).

_____ Midwife / doctor

_____ WIC

_____ Medi-Cal enrollment

_____ CalWorks enrollment

_____ Food Pantry

_____ Baby Supplies

_____ Education

_____ OTHER:____

3. How frequently would it have to come to be useful? What type of time-window is sufficient to be useful?

Additional Comments:

COMMUMITY CENTER FOR PREGNANCY AND YOUNG FAMILIES

Description

A community center focused on providing support and services for pregnant individuals and their families.

Questions for You

1. How much do you like this idea? Rate it from 1-5:

1	2	3	4	5
**Don't love it**		**It's okay**		**Love it**

2. Which areas of the city would you **NOT** want this center to be based in? Put an **X** in these areas:

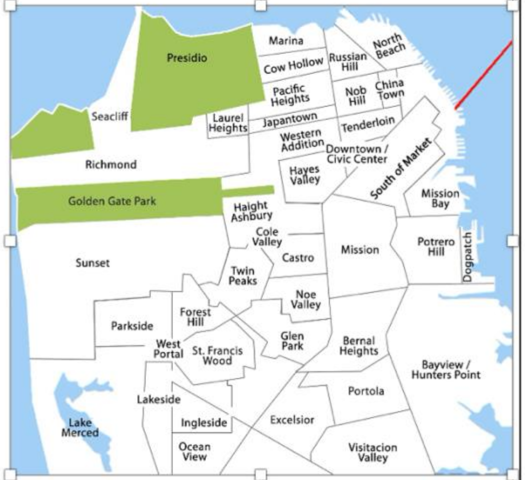

3. Circle the 5 things that this community center must have:

Medi-Cal consultation	Lactation
Calworks	Pediatrics
WIC	Community kitchen
Housing	Food pantry
Preschool studio	Yoga or dance
Navigators	Salon
Virtual Education	Gym/personal training
Educational Library	Childcare
Prenatal care	

Where would you want this to be? Circle one:

Zuckerberg San Francisco General Hospital　　　In the community

OTHER:____________

Would you prefer to have a mobile unit with limited services come to you, or a community center with all services under one roof?

Community Center　　　Mobile Unit

What did we miss?

Overall Feedback on all prototypes:

Iterated (13 item) version:

First, we'd love to hear from you:
How old are you? _______________
Gender that you identify with (circle one): Male Female Other
Race/ethnicity/culture you most identify with: _______________
Have you ever been on Medi-Cal/Medicaid insurance? Circle one: YES NO
Have you been pregnant before, or been a partner to someone who was pregnant? Circle one:
YES NO
If you have been pregnant, were you on Medi-Cal/Medicaid insurance at that time ?
Circle one: YES NO
SF Neighborhood you live in or consider home (or city, if don't live in SF)_________________

SERVICES THAT COME TO YOU

Description
A mobile unit that travels to your neighborhood with helpful services and offerings.

Questions for You
1. How much do you like this idea? Rate it from 1-5:

**1**	**2**	**3**	**4**	**5**
**Don't love it**		**It's okay**		**Love it**

2. Rank these services from 1 (most important) to 8 (least important).

_____ Midwife / doctor
_____ WIC
_____ Medi-Cal enrollment
_____ CalWorks enrollment
_____ Food Pantry
_____ Baby Supplies
_____ Education
OTHER:____

3. How frequently would it have to come to be useful? What type of time-window is sufficient to be useful?

Additional Comments:

COMMUNITY CENTER FOR PREGNANCY AND YOUNG FAMILIES

Description

A community center focused on providing support and services for pregnant individuals and their families.

Questions for You

1. How much do you like this idea? Rate it from 1-5:

1	2	3	4	5
**Don't love it**		**It's okay**		**Love it**

2. Would you prefer to have a mobile unit with limited services come to you, or a community center with all services under one roof?

Community Center

Mobile Unit

3. Circle the 5 things that this community center must have:

Medi-Cal	Calworks	WIC Services	Pregnancy classes
Pediatrics	Prenatal care	Breastfeeding support/help	
Navigators	Housing	Food pantry	Educational library
Childcare development center	Childcare	Virtual Education	
Community kitchen	Yoga or dance studio	Salon	
Gym/personal training			

4. Where would you want this to be? Circle one:

Zuckerberg San Francisco General Hospital In the community

OTHER:_______________

5. Which areas of the city would you **NOT** want this center to be based in? Put an **X** in these areas:

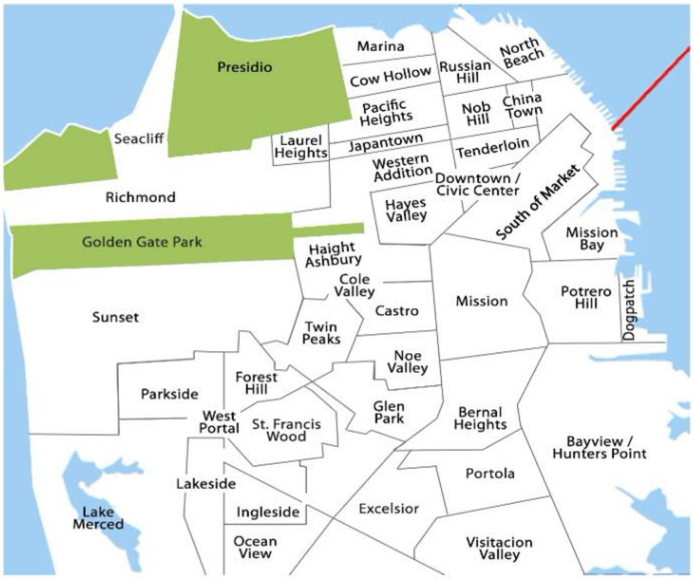

Additional Comments:

BUILDING COMMUNITY WITH YOUR CARE TEAM

Description

Get to know your doctors and midwives in a setting outside the clinic, to build trust and relationships.

Questions for You

1. How much do you like this idea? Rate it from 1-5:

1	2	3	4	5
**Don't love it**		**It's okay**		**Love it**

2. What would you want to see at these gatherings?

3. What would help you build trust with your healthcare provider?

Additional Comments:

SUPPORT SISTER

Description

A person working in the clinic who has already gone through this experience, and is there to guide, support and get you what you need throughout your pregnancy.

Questions for You

1. How much do you like this idea? Rate it from 1-5:

1	2	3	4	5
**Don't love it**		**It's okay**		**Love it**

2. What does a "support sister" look like to you? What characteristics does this person have?

Additional Comments
